# Altitudinal limits of Eastern Himalayan birds are created by competition past and present

**DOI:** 10.1371/journal.pone.0217549

**Published:** 2019-07-10

**Authors:** Gautam S. Surya, Timothy H. Keitt

**Affiliations:** 1 Department of Integrative Biology, University of Texas at Austin, Austin, TX, United States of America; 2 Wildlife Conservation Society, Bronx, NY, United States of America; University of Colorado, UNITED STATES

## Abstract

The degree to which interspecific competition structures diverse communities is an oft-debated topic. An approach to answering this question is to examine spatial patterns of coexistence among putatively competing species. The degree to which interspecies competition predominates in a community can have important effects on our ability predict the response of that community to perturbations, most notably climate change, when shifting species’ ranges may result in novel species assemblages. We present a study on the avifauna of the Eastern Himalayas. We hypothesize that in a community where competitive interactions predominate, there will be a relationship between pairwise altitudinal overlaps and morphological differences between species. Moreover, we hypothesize that both morphological traits and altitudinal traits depart from a Brownian motion evolution model, resulting in species trait covariances having a phylogenetic component. We find a significant relationship between morphological dissimilarity and altitudinal overlaps of species pairs. We also find that closely related species are significantly more altitudinally stratified than a null model would predict. However, as more distantly related species pairs are included in the analysis, this pattern disappears, indicating that competitive interactions predominate only in closely related species. This is further suggested by the fact that altitudinal ranges themselves are phylogenetically overdispersed at the genus level, as are morphological traits. This effect disappears when the entire phylogeny is examined, with morphology and altitude being phylogenetically underdispersed. Model fitting suggests that individual clades have evolved towards local clade-specific fitness peaks, while within-clade results show evidence of niche partitioning. We interpret these results as a tension between competition on shorter time scales and selection on longer time scales, where competition forces closely-related species away from fitness peaks in order to allow for niche separation and hence coexistence, suggesting that this effect is partially responsible for the recent diversification of Eastern Himalayan avifauna.

## Introduction

The strength of interspecific competition is an oft-debated topic in community ecology, particularly as it pertains to high-diversity ecosystems. Interspecific competition has long been thought to reduce diversity through competitive exclusion [1–4]. The principle of competitive exclusion generally states that stable coexistence between two species cannot occur if those species share the same niche, in the absence of other mechanisms such as dispersal limitations [5]. High-diversity areas pose a challenge to this idea, as they possess seemingly far fewer niches than species [6–8].

Niche differentiation is a process in which new niches can be created for species in a community by partitioning existing niches. This in turn can result in disruptive selection, causing character displacement [9,10]. Through the creation of new niches in a community, resulting in greater access to exclusive resources, high diversity can exist stably in the presence of strong competitive interactions. An alternative paradigm, the unified neutral theory of biodiversity, has also been proposed as an explanation for species coexistence in high-diversity ecosystems. This alternative postulates that all individuals of all species in the same trophic level in an ecosystem are ecologically equivalent and therefore competitively neutral, with coexistence maintained by dispersal and stochastic local extinction and colonization events [11,12].

Neutral theory has generated a significant amount of controversy, and some tests of its predictions have been negative (see e.g. [13–15]). There is little empirical evidence to support the notion of competitive neutrality between individuals of all species in a community [16]. Nevertheless, it appears that neutral theory can provide a reasonable approximation of the patterns seen in certain systems (see e.g. [17–19]), although this does not preclude other explanations. Moreover, a weaker extension of neutral theory, which postulates that species in a community have equivalent average fitness, is well supported in stable communities [16]. Additionally, nearly neutral communities, where single species violate neutrality, may also give rise to similar patterns [20].

The Eastern Himalayas are a global biodiversity hotspot [21,22]. In particular, they have the highest passerine bird density on the planet [22]. Invasions from Southeast and Central Asia, followed by vicariance, are thought to have built up the avian biodiversity in this region, with most terminal splits dating pre-Pleistocene [23,24]. This implies that the avian community in the Eastern Himalayas has likely been static in composition for a long period of time. Birds are a tractable study system as they are numerous and conspicuous, they can be readily identified to the species level in the field, and their taxonomy has been well established [25]. Therefore, the Eastern Himalayan avifauna offers an excellent system to test the degree to which competitive dynamics might affect structure a diverse community on both short-term and evolutionary timescales. There is some *a priori* reason for expecting competition to be a strong force in this system. Studies on individual clades have indicated that competition is important [26–28]. Moreover, a recent study by Srinivasan et al 2018 assigned a strong role to competition in determining altitudinal range limits [29]. On the other hand, another recent study, Elsen et al 2017, suggested that abiotic variables play the primary role in setting altitudinal range limits, with the effects of biotic interactions such as competition being comparatively small [30]. This may suggest that neutral dynamics predominate in the system. However, the latter study did not rule out the possibility that narrow ranges of thermal tolerance may themselves have evolved as a consequence of niche differentiation over evolutionary timescales.

In this study, we adopt a coexistence-based approach to analyzing the strength of competitive species interactions in Eastern Himalayan avifauna. Patterns of species coexistence have been used as a proxy for testing competition for many years since the classic studies by Diamond [31,32]. With the increasing availability of phylogenetic data, an equivalent method is the age-range correlation, where the range overlaps of species are correlated with their phylogenetic relatedness–a negative relationship is assumed to indicate the presence of competitive interactions between them that have mediated secondary contact post-speciation [33]. However, neither of these approaches can be applied naïvely. Null models are required to properly interpret co-occurrence data [34,35], with pairwise analyses being suggested as a preferable alternative [36]. On the other hand, age-range correlation analyses can be affected by signals from allopatric speciation alone, in the absence of any secondary contact, though this is mitigated by the fact that most Eastern Himalayan species are most closely related to species outside the Eastern Himalayas [22,37]. A careful approach accounting for both evolutionary dynamics and phylogenetic confounding variables is required to correctly understand the implications of community-wide co-occurrence data.

If, as posited by Srinivasan et al, competition is a critical driver of range limits in this system, then following post-speciation secondary contact, coexistence could be maintained by altitudinal stratification, with little spatial overlap between closely related species. Alternatively, species pairs could diverge along functional trait axes, resulting in character displacement and allowing for spatial overlap. In this study, we therefore hypothesize that competition would result in a significant positive correlation between pairwise spatial overlap and morphological differentiation, with more morphologically distinct species pairs co-occurring more frequently. Moreover, it is often stated that competition is stronger between closely related species, but this is seldom explicitly examined. We therefore hypothesize that the strength of the correlation between morphology and spatial overlap will decrease when examining distantly-related species pairs. We also anticipate that measures of phylogenetic dispersion for both traits and occupied altitudes will show positive phylogenetic autocorrelation (i.e. underdispersion of traits) when examining the phylogeny of the entire breeding bird community, as closely related species converge on fitness peaks suitable for a given life history. Finally, we anticipate that examining the same measures of phylogenetic autocorrelation at the scale of individual genera should show the opposite pattern, due to competition forcing species away from optimized traits to allow for coexistence between closely related species.

A better understanding of the preponderance of niche versus neutral dynamics in a community can lead to a better understanding of species’ range limits. The mechanistic limitations of a species’ range arise from a complex set of evolutionary and ecological factors [38–40]. Species are subject to physiological limitations that in turn may have arisen from purifying selection due to competition [41]. By contrast, interspecies competition can help set the range limits of species even in the absence of physiological limitations [29,30,42]. Similarly, dispersal may affect local adaptation at the edges of a species’ range in the absence of any biotic factors [43]. The presence of parasites and pathogens can also impose selective pressures reinforcing range limits [44].

Understanding the mechanisms behind the maintenance of range limits is critical in understanding how spatial ranges may change in response to changing environments. This is especially pressing in the context of climate change, a potential major driver of such novel assemblages [45]. Climate change has already caused shifts in species’ ranges, and this is expected to accelerate over the next century [45–47]. As species move around on a continental scale, species currently found in allopatry or parapatry may become increasingly sympatric. It is critical to understand whether these species may then exclude each other due to competition, or whether they will be able to stably coexist. Species whose ranges limits are partially determined by competitive interactions may suffer increased losses if climate changes forces the abiotic components of their niche into greater geographical overlap. Alternatively, species may benefit from the displacement of a competitor. Moreover, species whose range limits are determined by biotic rather than physiological constraints may be better able to adapt rapidly to climate change. Alternatively, species subject to hard physiological limits due to selection for a narrow range of climate tolerances may be unable to adjust to changes in climate, and therefore experience range shifts. The ability to better predict this response has immediate and important consequences for the protection of biodiversity in the region.

## Methods

### Surveys

**Fig 1:**
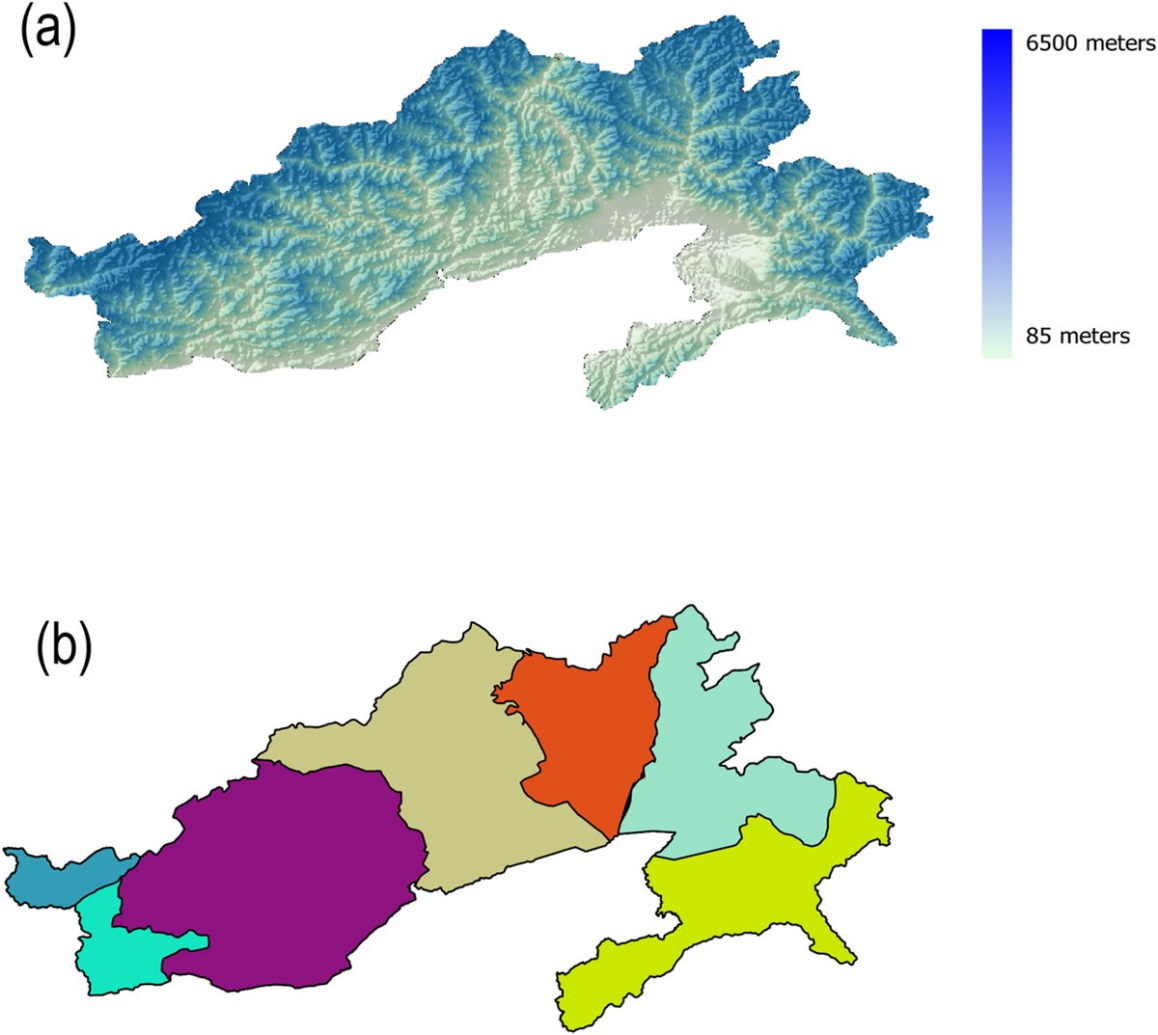
Maps of the study area. (a) ASTER digital elevation model (DEM) of Arunachal Pradesh draped over a hillshade map, courtesy NASA/JPL-Caltech. Regular survey points are in black; locations where species localities were collected opportunistically are in red. (b) Subregions used for the purpose of this study. From west to east, dividing lines between subregions are the Sela Massif, Kameng River, Subansiri River, Siang River, Dibang River and Lohit River. Arunachal Pradesh outline courtesy the Data{Meet} Community Created Maps of India project; subregions were digitized in QGIS software using the DEM above as a guide.

Surveys were conducted in the state of Arunachal Pradesh, NE India (Fig 1A) from 2013–2015. The coordinates for all survey points fell between 27.006 and 28.622 degrees North, and between 91.985 and 96.545 degrees East. Surveys were carried out during the monsoon (May-July), which is the breeding season for the majority of the regional avifauna. 201 survey points were selected at elevations ranging from 118–4354 meters in altitude; areas both along and away from roads were included, as were points from both protected and non-protected areas. Efforts were made to randomize across vegetation types at particular altitudes. At each survey point, two observers compiled comprehensive lists of all species seen/heard at ten minute intervals. Most sites were visited multiple times in order to assess detectability; sites were visited for an average of 9 intervals (range 1–30). In all, surveys totaled 328 hours of effort. Point localities for some species, generally those of conservation concern, were also collected opportunistically. We were able to record 375 species, 215 of which were recorded at a minimum of five survey points (see S1 Table in the Supporting Information).

### Modelling elevational ranges and range overlaps

**Fig 2:**
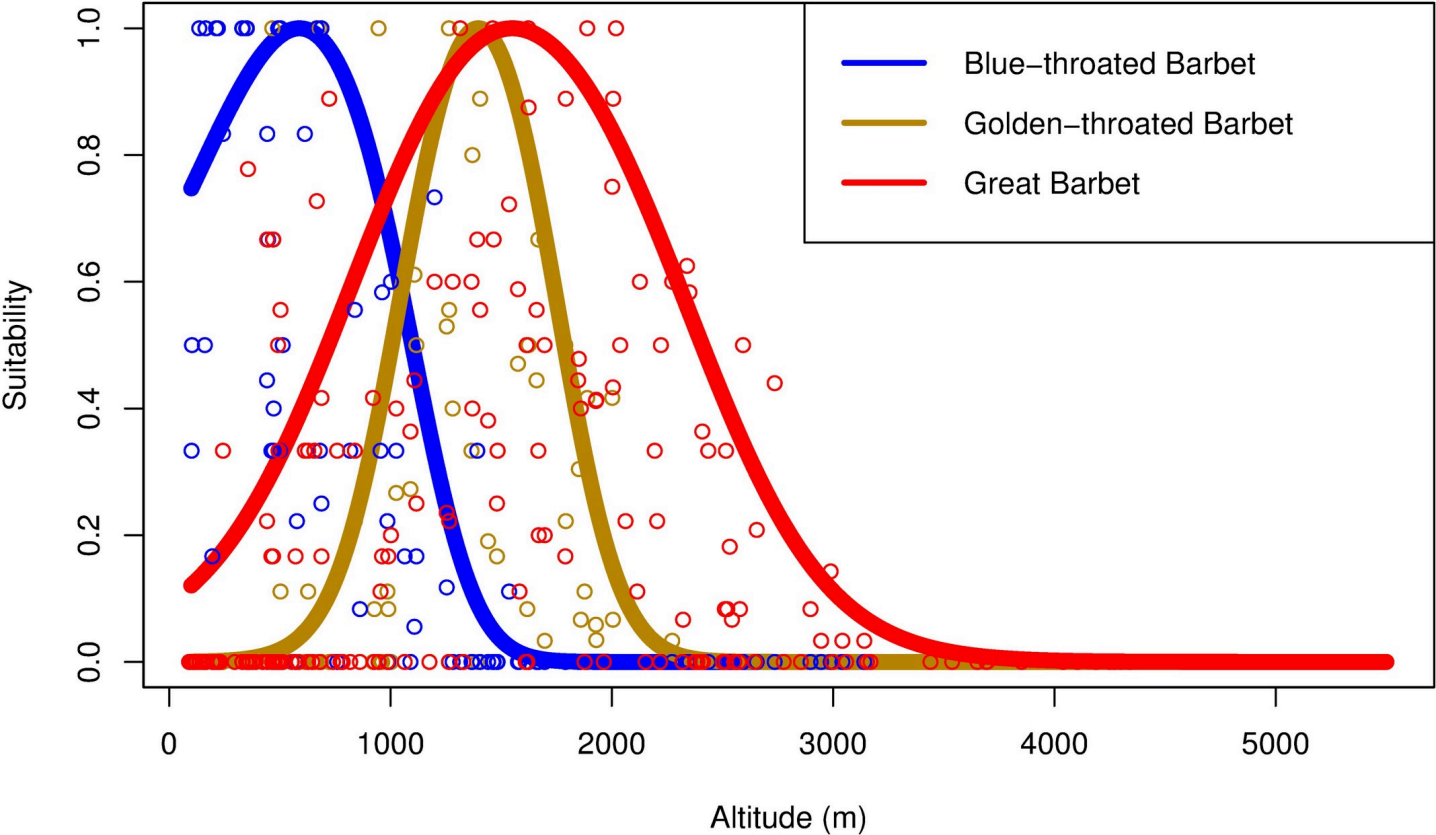
Predicted habitat suitability relative to altitude for three species of *Psilopogon* barbet. Solid lines represent generalized additive model (GAM) predictions based on survey data; open circles represent the proportion of surveys at a given location in which each species was detected. Notably, Blue-throated and Golden-throated Barbet are similarly sized and segregate altitudinally; Great Barbet, which is much larger, overlaps broadly with both.

For each species at each point, the data consisted of a series of successes (1) or failures (0) to detect the species at each interval at each survey point (see S2 Table in the Supporting Information for the full dataset of species detections). We modelled the elevational range using a generalized additive model (GAM) with a logit link function on this binomial data; we constrained the fit to a cubic smoothing spline with three knots, as elevational ranges are generally unimodal. The GAM output was then normalized to have a maximum value of 1. Pairwise elevational overlaps were taken to be ratio of the intersection to the union for the output curves (Fig 2); these were calculated using numerical integration. For each species, we also determined the central elevation as the 50^th^ percentile value of the GAM curve.

A common critique of studies using coexistence to make ecological inferences is the lack of a suitable null model [34,37]. Through chance alone, species occupying wide ranges of elevation will overlap more than species occupying narrow ranges of elevation. We therefore generated a null expectation for the elevational overlap for a given pair of species based on the size of their altitudinal preferences. It is known that bird diversity in the Eastern Himalayas relates to elevation [22]. We used a smoothing spline to estimate this relationship based on the average number of species observed per ten minute interval at each site. For both species being compared, we used this relationship as the basis of a Monte Carlo draw to provide a randomized central elevation. We then shifted the actual altitudinal preferences of both species upslope or downslope to align it with the randomized central elevation and calculated the resulting altitudinal overlap. This was performed 100 times for each species pair, with the calculated altitudinal overlaps then averaged to create a null expectation. We then subtracted this value from the observed elevational overlap. A value of zero indicates a species pair that was observed to overlap in altitude as much as would be expected from chance alone given the size of the altitudinal bands they occupy; values below and above zero indicate less overlap than expected from chance and more overlap than expected from chance respectively. We refer to this as the range-size corrected elevational overlap (RSCEO).

**Fig 3:**
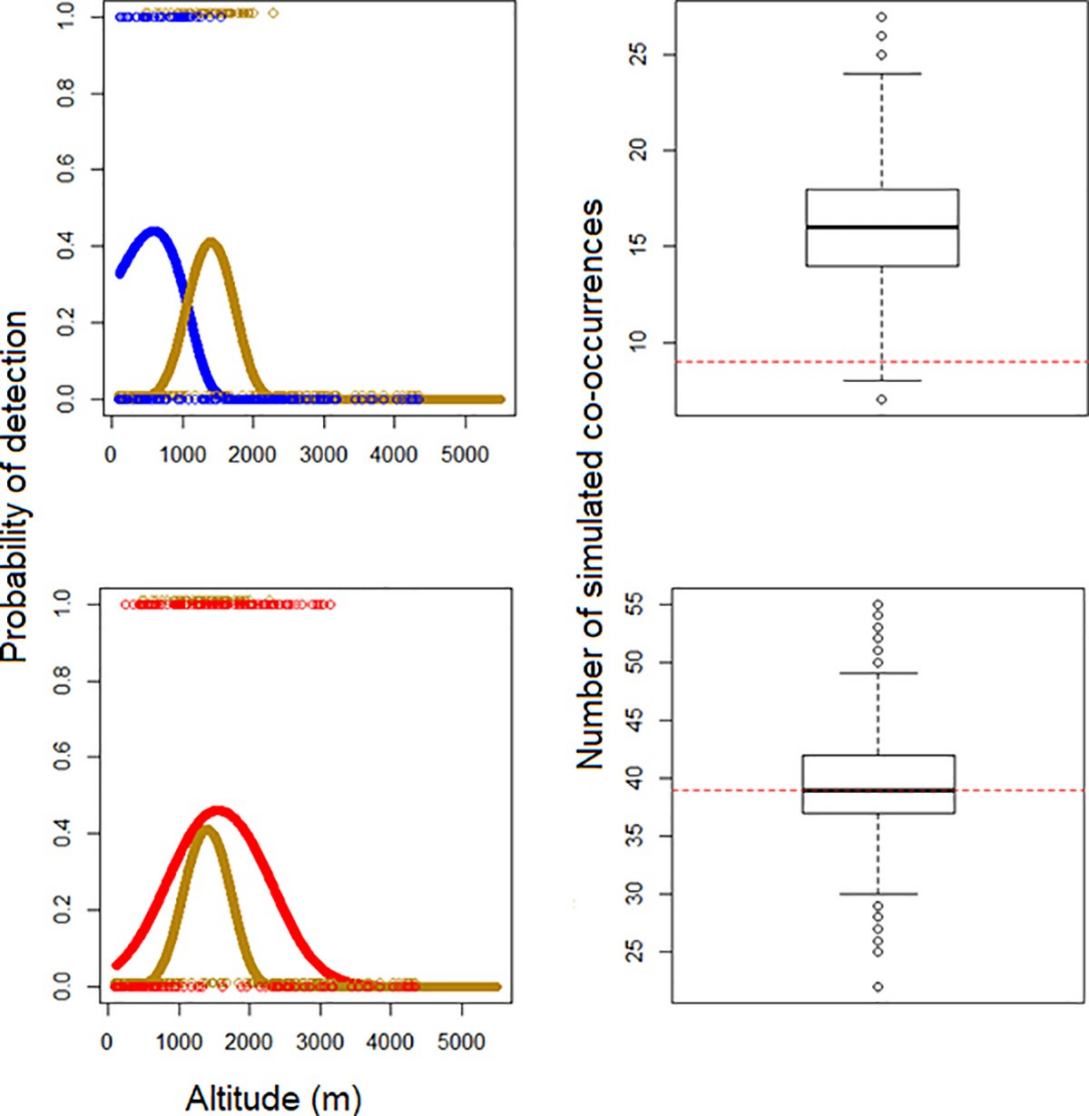
Simulated vs. actual co-occurrence in three species of *Psilopogon* barbet. (a) Golden-throated (yellow) and Blue-throated Barbet (blue). (b) Golden-throated (yellow) and Great Barbet (red). Left figures show relationship between habitat suitability and elevation for each species; open circles represent presence/absence at each survey point. Right figures show box-and-whisker plots of simulated co-occurrences across 5000 simulations; red dashed line represents the actual number of co-occurrences during surveys. Golden-throated and Blue-throated Barbets are similar sized; Great Barbet is much larger.

Moreover, elevational overlap alone is not necessarily indicative of sympatric coexistence. It is possible for a given species pair to overlap broadly in altitude, yet seldom coexist at a smaller spatial scale, which would be evidence of competition not captured by altitudinal overlap alone (Fig 3). We therefore developed a simulation model to determine the expected number of points at which a given pair of species would be found together assuming no competition, based on the GAM models above. The non-normalized GAM output is probability that a species would be detected at a given elevation during a single time interval during our surveys. We used this probability as the basis to simulate survey data at each survey point using a Monte Carlo approach; the length of the series at each point was based on the number of intervals that site was visited. We collapsed this series of detections into a single presence/absence value, with a species being considered present if there was at least one simulated detection at a site. This was done independently for both species being compared. The simulated presence-absence data for both species was then compared site by site in order to generate a predicted number of co-occurrences, and then compared to the actual number of points the species was found together at during our surveys. From each set of simulated survey data, we calculated a competition coefficient:

$$c_{i}=\sqrt{\frac{\mathrm{Actual}\;\mathrm{co}‐\mathrm{occurences}}{\mathrm{Expected}\;\mathrm{co}‐\mathrm{occurences}}}$$

The final value for the competition coefficient, *c*, was averaged over the *c*_*i*_ obtained from 5000 simulations. If the number of expected co-occurences from a simulation was 0, the value of c_i_ for that simulation was taken to be 1, a conservative choice. We multiplied the elevational overlap by *c*; we refer to this as spatial co-occurrence (SC).

Our study area, Arunachal Pradesh, has several major rivers and ridges. It is known that some of these features present geographical dispersal barriers. For example, the Sela Massif forms a border between the sister species Rufous Sibia *Heterophasia capistrata* in the north/west, and Beautiful Sibia *H*. *pulchella* in the south/east [48,49]. Similarly, the Rusty-throated Wren Babbler *Spelaeornis badeigularis* is found to the west of the Dibang River, while its sister species, the Rufous-throated Wren-Babbler *S*. *caudatus* is found to the east [48,49]. In order to avoid false negatives in our data, we divided Arunachal Pradesh into several subregions (Fig 1B) based on a set of rivers and high-altitude ridges. From west to east, the dividing lines were taken to be the Sela Massif, the Kameng River, the Subansiri River, the Siang River, the Dibang River, and the Lohit River. We determined whether each species was present or absent in each subregion based on a combination of field guide maps along with our own surveys. In subregions where a species was not present, the survey data was taken to be NA rather than a 0 for GAM fitting; we also omitted pairwise comparisons of congeners not found sympatrically in at least one subregion.

### Trait and phylogenetic data

We had two main sources of trait data. Measures of head length, body length and overall length for all species were obtained from Rasmussen and Anderton 2005; measures of bill length/width/depth, tarsal thickness and wing chord for most passerine species were obtained from the supplemental material of Price et al 2014. These traits are known to correspond to functional aspects of a bird’s ecology and foraging behaviour [50]. The set of morphological trait measurements is available in the Supporting Information, S3 Table.

For phylogenetic data, we relied on global bird phylogeny created by Jetz et al [25]. This phylogeny was generated by assigning species to a set of crown clades and generating trees for these clades, then combining them onto a set of backbone clades [25]. We downloaded a sample of 1000 trees from a pseudo-posterior distribution, including all species found during our surveys as tips [25] (trees available in Supporting Information, S1 Tree and S2 Tree). From these, we calculated the pairwise patristic distance between all species. These distances were used to select a set of congeneric sister species (CSS) found during our surveys, as it is generally thought that the competition is strongest between closely related taxa; species belonging to a genus with only two representatives in our surveys were considered a part of the CSS set. It is worth noting that the sister taxon for the majority of Eastern Himalayan passerine species is extralimital [22]. Among the 210 species detected at 5 or more points, we were able to form 35 CSS pairs. From these, we excluded the aerial insectivores *Delichon nipalense* and *Delichon dasypus*, as their habit of foraging at high altitudes makes it difficult to interpret survey occurrences as being representative of their breeding ranges. The full list of 34 CSS pairs is available in the Supporting Information, S4 Table.

We based the taxonomy for this study on the IOC World List v. 7.1; all datasets describe above were reconciled to this taxonomy. One species pair, *Psittiparus ruficeps* & *P*. *bakeri*, was not found in the phylogenetic data and was therefore excluded from those analyses; the two taxa were considered conspecific unt recently and are currently thought to exist only in allopatry.

### Statistical and phylogenetic methods

**Fig 4:**
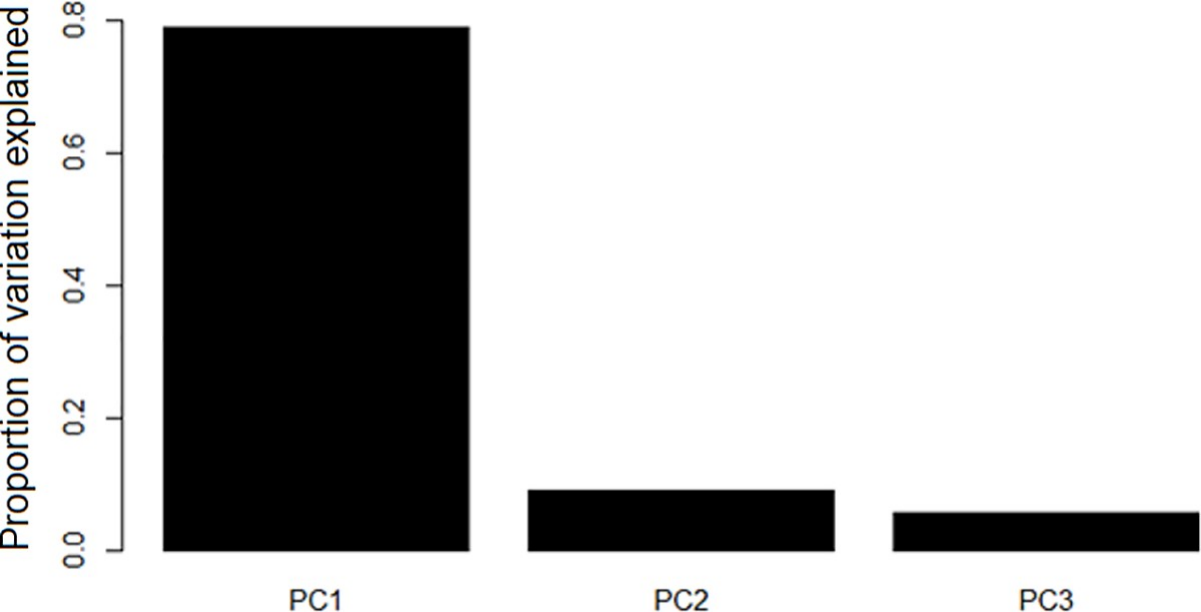
Principal component analysis of morphological variance. PC1 explains the vast majority of the data, 78.9%; the first three principal components explain 93.6% of the data. PC1 broadly corresponds to overall body size; PC2 corresponds to large-billedness, and PC3 corresponds to long-tailedness.

The available morphological variables were highly collinear, making direct interpretation complex. To overcome this, the morphological variables were log-transformed and a principal component analysis (PCA) was performed (Fig 4). The first component, corresponding to overall size, explained over 78% of the variation in the data; the first three explained over 93% of the variation. Missing passerine data was predicted using the PCA fit from the passerine species in the Price et al dataset, based on values in the Rasmussen and Anderton dataset. We tested the relationship between morphological dissimilarity and range overlaps by regressing measures of range overlaps against the Euclidean distance between species in PC space.

Additionally, we wanted to test the oft-asserted notion that the strength of competition is stronger among closely related species. We did this by applying varying time cutoffs to the phylogeny. For each individual cutoff, we selected species pairs with a most recent common ancestor (MRCA) more recent than the cutoff. We then calculated the correlation between range overlaps and morphological similarity for those species pairs. Finally, we looked at the relationship between the cutoff chosen and the correlation between range overlap and morphological similarity.

We examined the effects of phylogenetics on traits using several methods. Moran’s I is a measure of spatial autocorrelation, which gives the relationship between a matrix of pairwise differences of some quantity and a matrix of pairwise spatial distances between them [51]. However, it can be extended to use in a phylogenetic context by replacing spatial distances with phylogenetic distances [52]. Positive values of Moran’s I indicate that closely related species are closer in trait space than would be expected; we generated the null expectation for Moran’s I by using Brownian Motion (BM) simulations on the consensus phylogeny. Abouheif’s C_mean_ is a similar test that uses a distance matrix where the major diagonal can take on nonzero values [53]. We calculated Moran’s I and Abouheif’s C_mean_ for morphology by assigning the values of PC1-PC4 as continuous traits. We calculated these measures for the altitude occupied by treating the central altitude as a quantitative trait, and creating a pairwise distance matrix accordingly. To test whether phylogenetic uncertainty could affect these results, we calculated Moran’s I and Abouheif’s C_mean_ for 100 randomly selected trees individual phylogenetic trees, as well as for our consensus tree.

### Software used

All analyses, including simulation models, were carried out in R [54]. In addition to the base packages, we used functions from ape, adephylo, geiger, missMDA, mgcv, phylobase and phytools [54–61]. Basic GIS processing (e.g. extracting the elevation of each survey point from a digital elevation model) was accomplished using QGIS v 2.18 [62].

## Results

For our 34 congeneric sister species pairs, we found that neither the competition coefficients nor the raw degree of elevational overlap were correlated with morphological similarity. This is in part due to the fact that species with little range overlap also have low calculated competition coefficients, since they are not expected to co-occur. Additionally, species that share a large portion of the elevational range may not co-occur at the scale of individual sites if they specialize on different microhabitat features (Figs 2 and 3).

**Fig 5:**
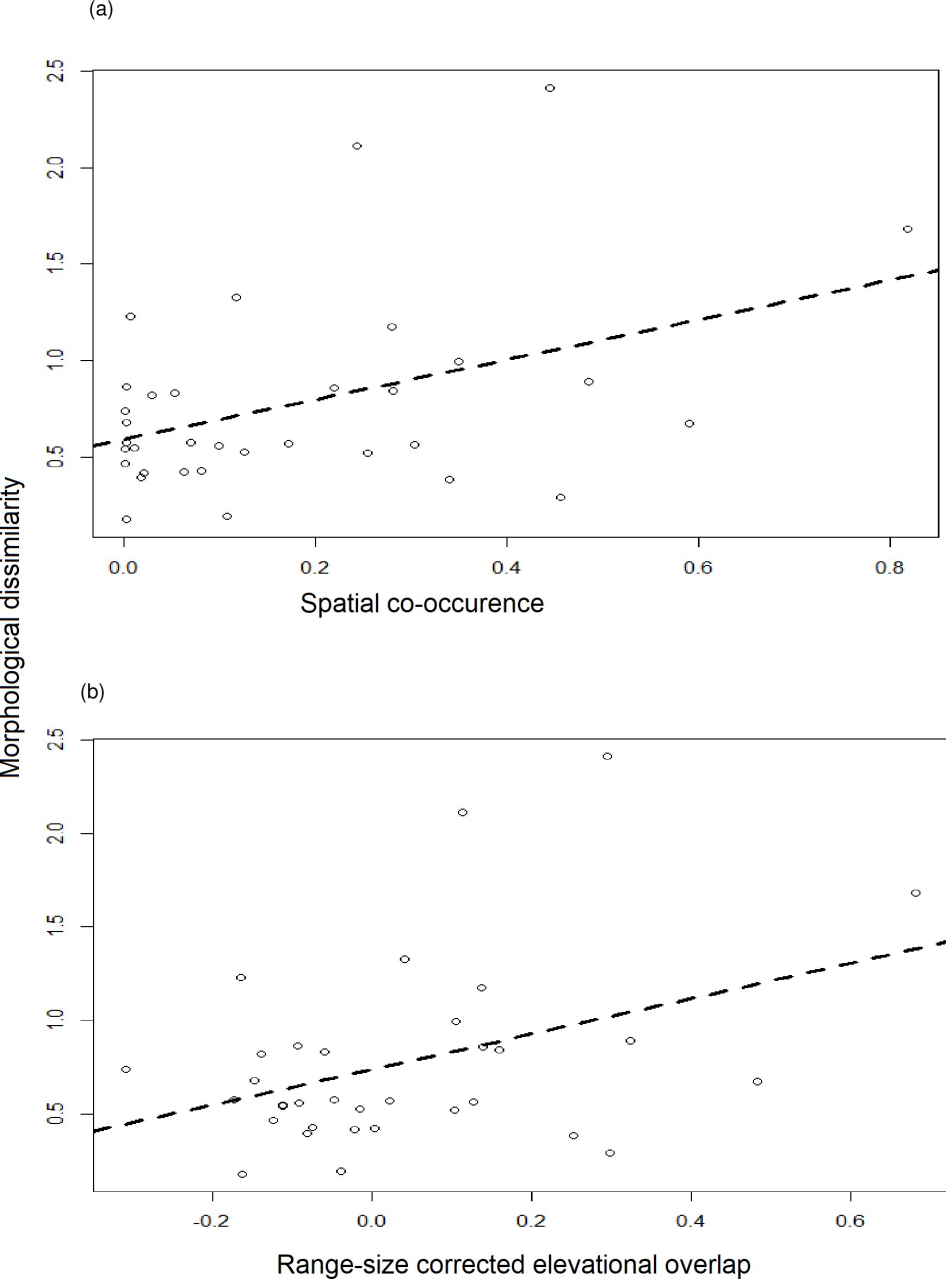
Relationship between morphological difference and elevational overlaps for sympatric congeneric sister species pairs. (a) Spatial co-occurrence; t = 2.59, df = 32, p = 0.014, r^2^ = 0.17. (b) Range-size corrected elevational overlap; t = 2.42, p = 0.021,r^2^ = 0.15. In both cases, morphologically dissimilar species are expected to have greater overlaps in range. Open circles represent individual sympatric congeneric sister species pairs; dashed line indicates linear regression fit.

However, when we examined range overlaps relative to our null expectations, we found a significant correlation with morphological differentiation. Spatial co-occurrence (SC) was found to be significantly correlated with the morphological difference between a species pair (Fig 5A; t = 2.59, df = 32, p = 0.014, r = 0.41). Species that differed more in morphology were expected to have more co-occurrence, and vice versa. This is persuasive evidence that competition plays a role in setting the altitudinal ranges of species at short temporal scales.

Additionally, range-size corrected elevational overlaps (RSCEO) were found to be significantly correlated with morphological differentiation, with morphologically distinct species showing greater than expected overlap (Fig 5B; t = 2.42, p = 0.021, r = 0.38). Moreover, observed elevational overlaps were significantly different from the null expectation based on elevational range size alone (paired student’s t-test; t = 2.06, df = 33, p = 0.047). It is worth noting that many species show greater than expected altitudinal overlap, i.e. a value of RSCEO greater than zero. This is likely due to the relationship between elevation and overall diversity, which peaks at mid-elevations and is thought to be driven by arthropod abundances [22]. As a result, there is a significant benefit to birds residing at this elevation, causing greater elevational overlaps in species pairs.

**Fig 6:**
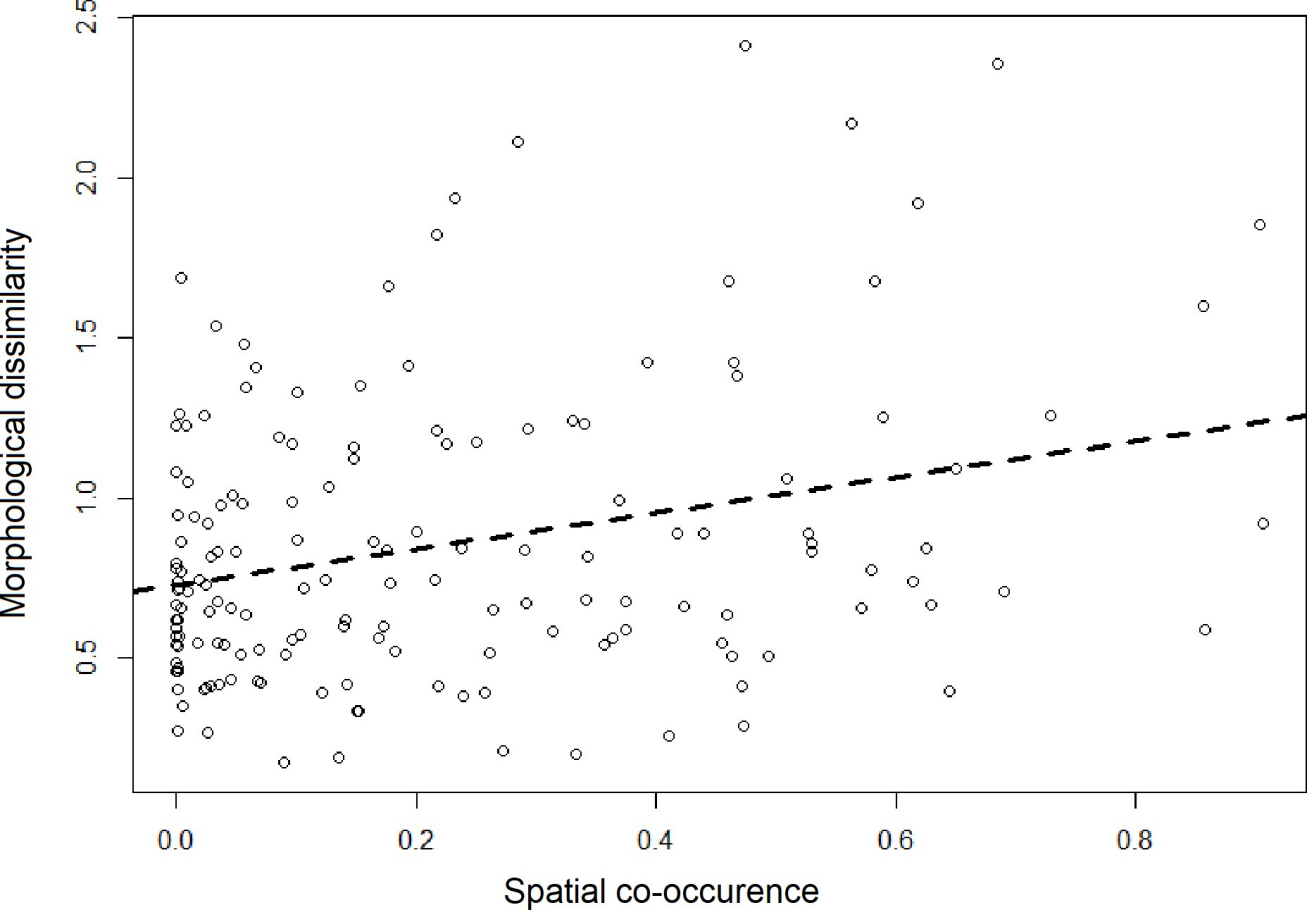
Relationship between morphological difference and competition corrected range overlaps for all congeneric species pairs. Open circles represent individual congeneric species pairs (159 pairs); dashed line represents the best-fit linear regression. t = 3.75, df = 157,p<0.005,r2 = 0.08.

**Fig 7:**
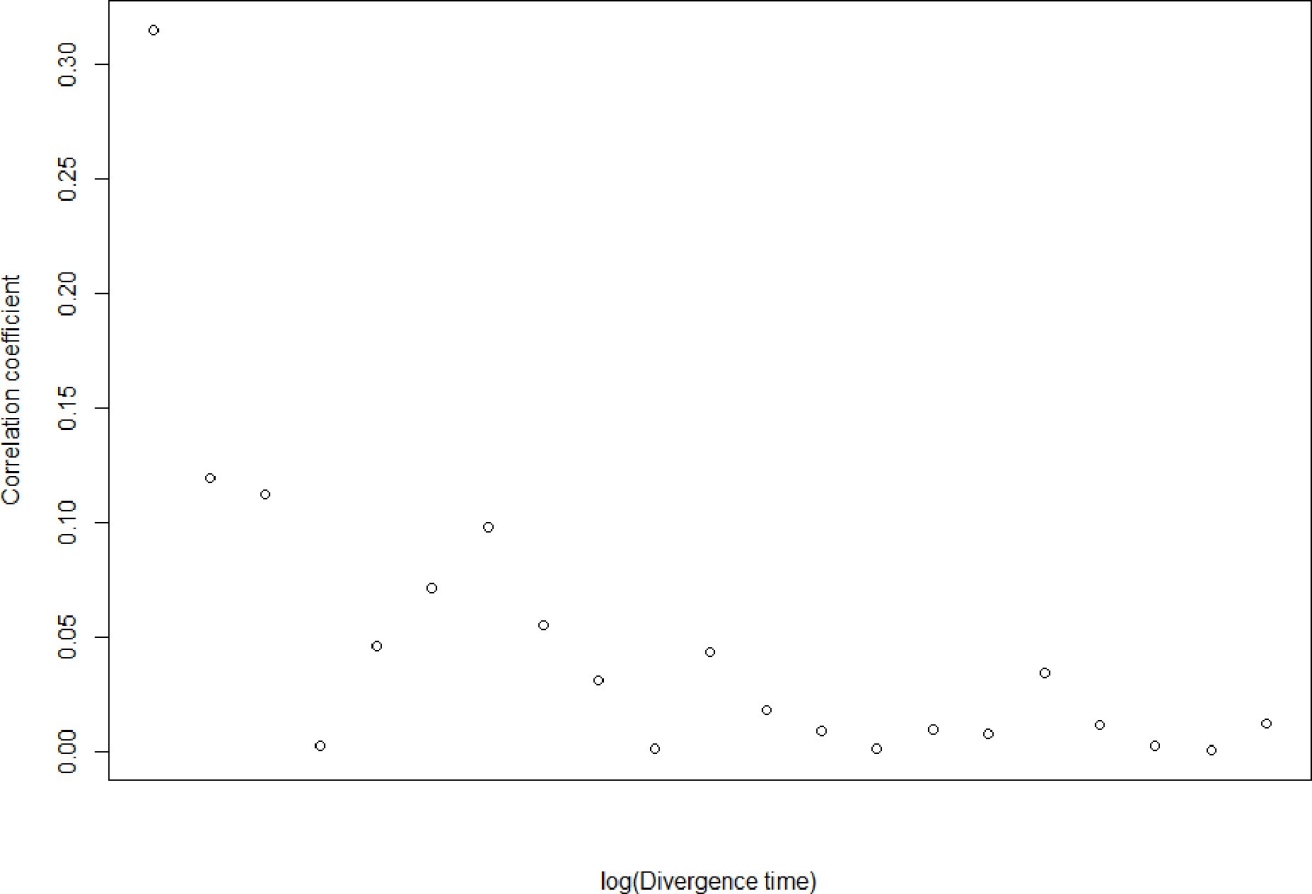
Relationship between divergence time between species, and the strength of the correlation between morphological difference and altitudinal overlaps for those species. Y-axis represents the r value for this correlation for all species pairs with a more recent MRCA than the X-axis value. As the X-axis increases, representing the inclusion of more distantly-related species pairs, the Y-axis value approaches zero. In other words, when including distantly-related species pairs, there is no correlation between morphological difference and altitudinal overlap. This implies that competition is only a factor between closely related species.

It is generally thought that the strength of competition is higher amongst closely related taxa. When considering all pairwise congeneric comparisons, and not just sister taxa, corrected range overlaps were still significantly negatively correlated to morphological difference, although the correlation was lower than when only sister species were considered (Fig 6; t = 3.89, df = 153, p<0.005, r = 0.29). As we examined more distantly related species, this correlation declined, providing empirical evidence to suggest that distantly related species do not appear to affect each other’s altitudinal ranges or functional traits (Fig 7); the correlation falls off quickly with divergence time.

**Fig 8:**
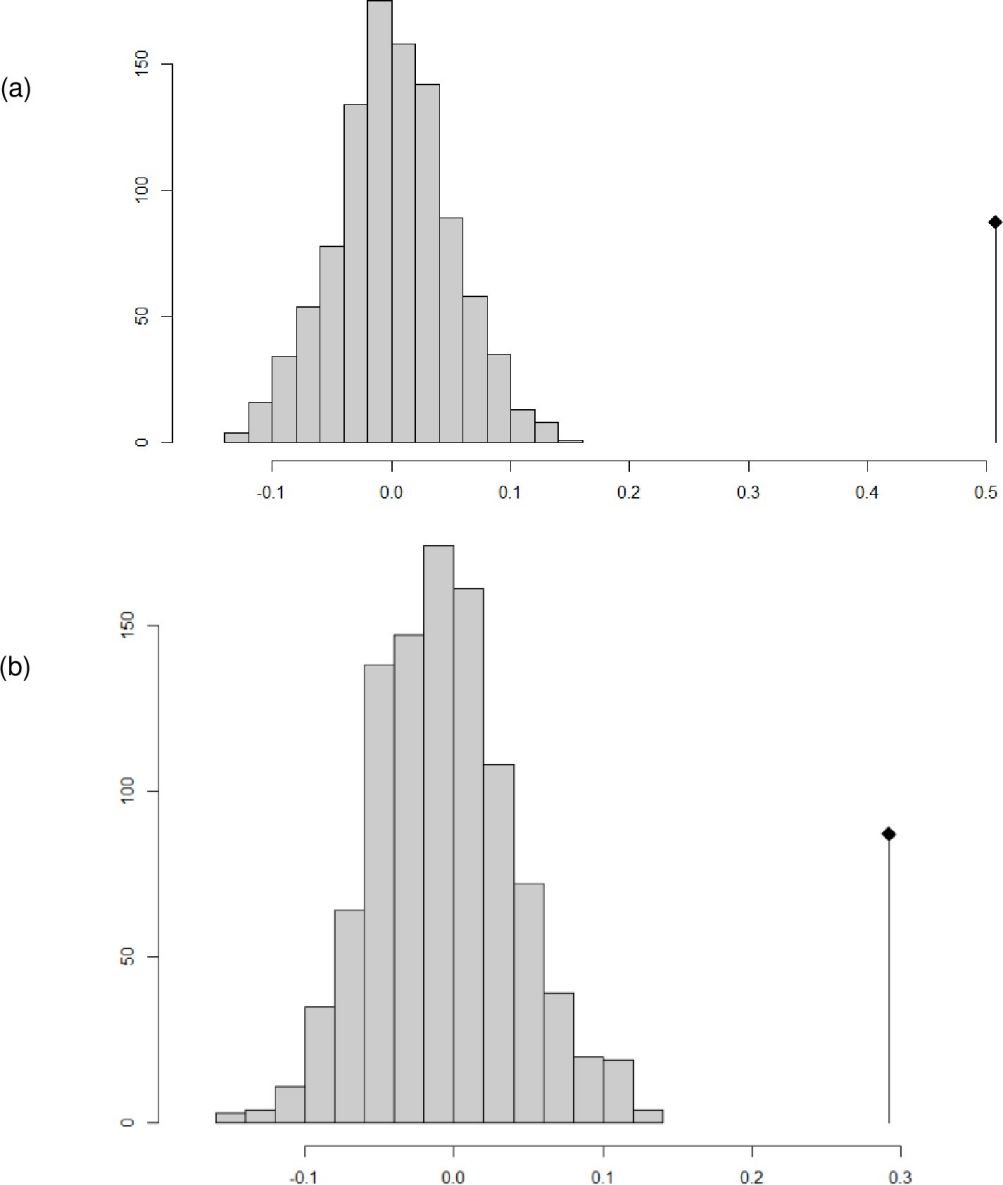
**Simulated and actual Moran’s I, all species, for (a) morphology and (b) elevation.** For morphology, the value for each species was taken to be its distance from centroid in principle component space after morphological variables. For elevation, the value for each species was taken to be the central elevation occupied by that species. Bars represent number of simulations out of a thousand resulting in a given value of Moran’s I; vertical line topped by diamond represents the observed value of Moran’s I. Positive values of Moran’s I indicate that species are more similar to their relatives than the null expectation.

We also found that when examining the entire phylogeny, there was evidence of non-random evolution of both morphological traits and central elevation (Fig 8). We created a consensus tree based on our set of 1000 phylogenies. We used the PCA values for morphology to calculate a distance from centroid for each species. For the consensus tree, observed values of Moran’s I and Abouheif’s C_mean_, were significantly underdispersed when compared to 1000 randomizations for both morphology and central altitude; both measures always deviated in the same direction. We also calculated Moran’s I and Abouheif’s C_mean_ for 100 randomly selected trees individual phylogenetic trees. In each case, the observed values of Moran’s I and Abouheif’s C_mean_ were significantly different from values generated by randomization, indicating that phylogenetic uncertainty is insufficient to alter the overall pattern. The underdispersion of Moran’s I and Abouheif’s C_mean_ indicate that related species are more similar than would be expected in the case of random noise. This pattern indicates that clades of species have evolved specialized morphologies corresponding to their ecological niches, foraging modes, etc. The observed underdispersion of the central elevation occupied by species is likely to be driven by the previously mentioned bias of species towards mid-elevations [22], resulting in species having more similar elevational ranges than would be expected by chance alone.

**Fig 9:**
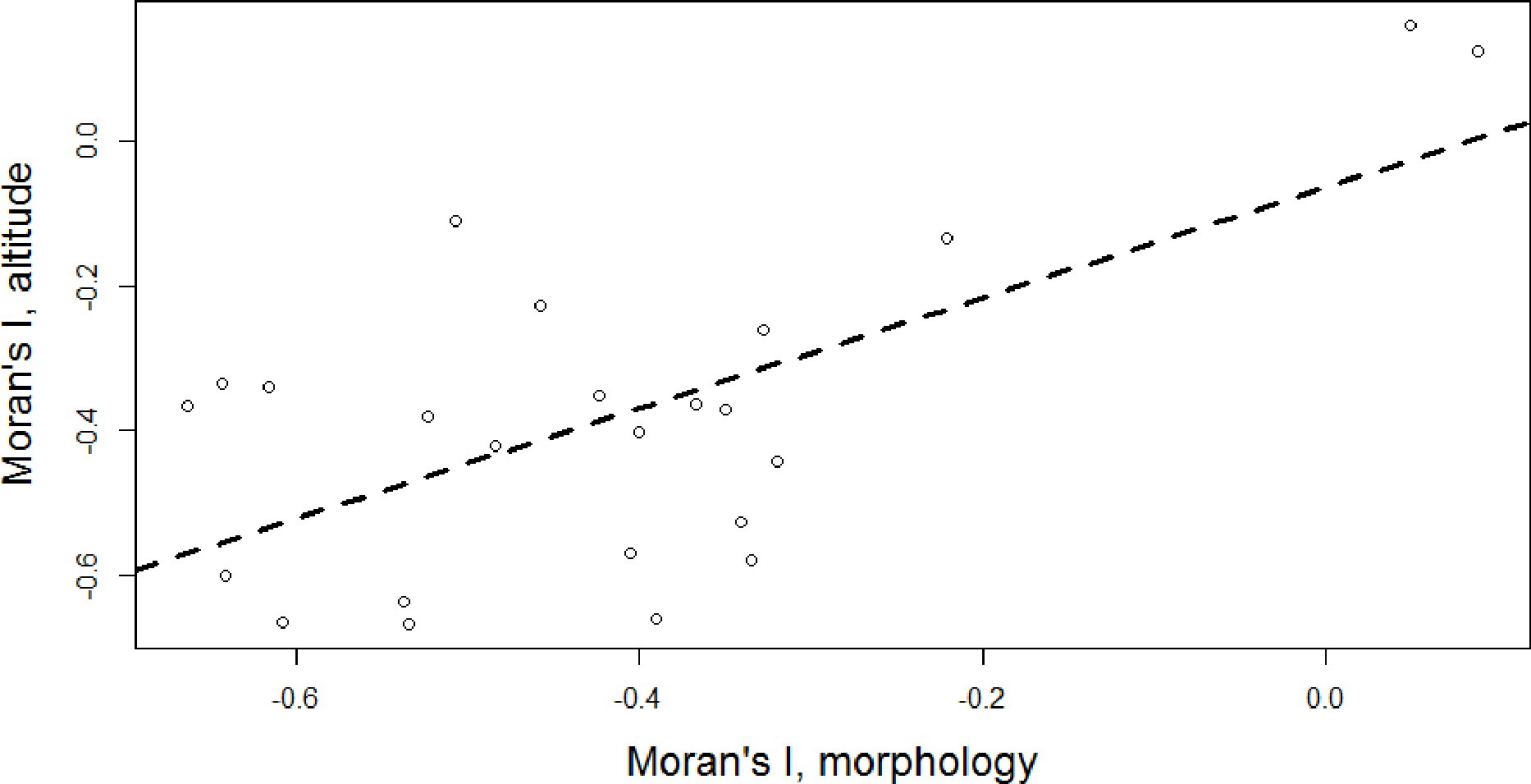
Relationship between Moran’s I for PC1 and Moran’s I for central elevation. Negative values of Moran’s I indicate overdispersed traits, though all results are not significantly different from a null expectation. Open circles represent individual genera; dashed line indicates best fit linear regression (t = 3.95, df = 22, p<0.001, r^2^ = 0.41).

Taken individually, genus consensus trees with at least 3 species exhibited overdispersion of traits (Fig 9). No single genus tree was significantly different from the null expectation, likely due to the relatively paltry number of species per genus. However, a sign test indicates that the median value for Moran’s I along both morphological and altitudinal axes is significantly less than zero (n = 24; for morphology, s = 2, p<0.0001; for altitude, s = 2, p<0.0001), providing support for the hypothesis of recent diversification in those traits. There was no indication of a tradeoff between divergence along each of these axes–in fact, there was a positive correlation between Moran’s I for morphological traits and Moran’s I for central elevation (t = 3.95, df = 22, p<0.001, r^2^ = 0.41). Qualitatively similar results were obtained from an analysis of a random sample of 100 individual trees; for all 100, values of Moran’s I clustered below zero for individual genera (p<0.05 for all 100 sign tests for PC1 and altitude).

## Discussion

Our results indicate that interspecific competition plays an important role in structuring the avian community of the Eastern Himalayas by causing altitudinal stratification. Our conclusions are derived from several lines of evidence, including phylogenetic relationships, trait data and spatial occurrence data. We interpret these results as occurring on multiple timescales. On a short timescale, there is a significant effect of morphological dissimilarity on the extent of spatial co-occurrences exhibited by a species. Moreover, closely related species pairs are more spatially segregated than would be expected under a null model. Among morphologically similar species pairs, the reduced co-occurrence is generated by two seemingly different mechanisms. Some species pairs have distinctly different elevational ranges and therefore co-occur rarely, with competition presumably playing a minor proximate role. Other species pairs appear to overlap broadly in elevational space, but exclude each other at smaller spatiotemporal scales and so are rarely found together, more indicative of direct competition. Further research is required to understand what explains these different strategies. We found that phylogenetic relatedness alone cannot explain it, as the age of a species pair’s most recent common ancestor does not predict the amount of co-occurrence relative to elevational overlap.

Our results also suggest that competition has played a role on longer timescales. Speciation driven by adaptive divergence can produce results that look facially similar to competition in a coexistence analysis [37]. We do not believe that speciation itself is responsible for the pattern we observe, as Eastern Himalayan avifauna has been built up through cycles of allopatric speciation and secondary contact, with the closest relatives of most Eastern Himalayan species located outside the region [22]. However, our results indicate that competition following secondary contact is a major driver of community assembly. We believe that species pairs that segregate to a large degree in elevational space, as discussed above, have evolved their ranges in response to competition occurring over a longer timescale. Our interpretation is bolstered by our findings that species’ morphological traits and elevational ranges do not seem to have evolved at random. When considering the phylogeny as a whole, morphological traits and elevational ranges are underdispersed, indicating conservatism around favored elevations and clade-specific morphologies. However, traits are divergent (overdispersed) across clades, indicating historical niche displacement along both morphological and thermal tolerance axes.

Older studies on passerine diversification in this region have emphasized the importance of interspecies competition and niche differentiation, particularly among individual clades [22,26,27]. Moreover, a recent community-wide analysis by Srinivasan et al showed competition to be a strong force in setting altitudinal ranges in the region [29]. However, another recent study by Elsen et al has called the importance of competition into question in favor of abiotic considerations such as temperature [30]. Our results align strongly with the Srinivasan et al study, showing that competition plays an important, if not exclusive, role in determining altitudinal range limits and structuring the community. Additionally, we suggest that this view can be reconciled with the Elsen et al study, as we find that differences in abiotic preferences are likely to be the result of past competition leading to niche displacement. Our work further indicates that there is an evolutionary tradeoff between selection for optimal morphologies corresponding to a clade-specific fitness peak, and selection for divergent morphologies within a clade that push species away from that peak. This can be seen in the fact that morphological trait values are more similar than would be expected from phylogenetic relatedness alone across the entire phylogeny, which may be explained by a convergence on a morphology suitable for general insectivory, the most common foraging mode for birds in the region [22]. Conversely, species in the same genus are more different in their functional morphological traits than would be expected from relatedness alone. The same applies to elevational ranges, which are a proxy for thermal tolerance. We observe a general preference for middle elevations, which have more abundant resources, but species are appear to have been forced away from these more favorable conditions by competitive interactions.

In cases where species pairs show similar morphologies and elevational preferences, yet do not co-occur on smaller scales, further work may elucidate what mechanisms are responsible. For example, studies on Andean birds have shown that aggressive responses to heterospecific songs are important in setting elevational range limits[63–65]. Additionally, there is some evidence that certain Sino-Himalayan species may display territorial responses to heterospecific song playback [66], although strength of response to different but closely related species is quite variable. Songs may also reinforce range limits of species pairs whose elevational ranges barely overlap, and in fact song traits may experience selection for this purpose [67]. However, it is worth mentioned that other Sino-Himalayan species show little response to heterospecific playback, and that field observations indicate that responses to playback are often highly species-specific [68,69]. Other mechanisms may include fine-scale niche partitioning within an altitudinal band as a response to structural habitat features, differential susceptibility to parasites and/or diseases, and differences in the time of daily foraging or other activity that allow for increased elevational overlap despite morphological similarity.

It is also worth mentioning that while our results indicate that competition plays a strong role in influencing species’ range limits in this system, it does not exclude additional contributory factors. Species’ range limits are known to be controlled by a variety of factors (see e.g. [38,39]). Some of these factors may mediate the way in which competitive species interactions play out across the landscape. For example, the species in this study have similar breeding phenologies linked to the onset of the monsoon season [49]. This synchrony in phenology likely exacerbates the effects of competition. Additionally, the altitudinal migrations undertaken by many species may affect the strength of competitive species interactions outside the breeding season. Further research is required to elucidate the strengths of these other mechanisms in contributing to the determination of elevational range limits.

In the coming century, anthropogenic climate change is expected to cause extensive perturbations to ecosystems and communities [70,71]. Several attempts have been made to predict the responses of avian communities to this challenge [72,73]. One of the major challenges of these efforts is predicting the plasticity of species in their abiotic tolerances. However, there is some evidence that birds in montane environments tend to track their preferred climate space over time [74]. Additionally, several species in the Eastern Himalayan avian community track their preferred climate space during seasonal migration [29]. Our results further indicate that thermal limits of many species are a product of continuing selection and are reinforced by interspecies competition. This suggests that many species may be challenged by climate change, as spatial range shifts may be countered by the presence of parapatric closely-related species. This may prevent species from shifting their ranges, thus subjecting them to rapid changes in their abiotic environment. Continuing selection on their abiotic preferences may hamper their ability to adapt to these changes.

Considerable effort has gone into the application of species distribution modelling (SDM) to estimate the change in species’ spatial ranges in the face of climate change [75]. However, these attempts are generally limited to consideration of changes in abiotic conditions alone [75,76]. Our results suggest that to accurately understand the effects of climate change on Himalayan avifauna, it is necessary to incorporate interspecies competition. If these competitive species interactions are omitted, predictions regarding the response of species to climate change are likely to be inaccurate. By correctly accounting for the effects of biotic interactions, we may more accurately predict the response of this remarkable system to the next century of rapid change, thus enhancing our ability to make critically required conservation decisions and helping to preserve it for future generations of researchers.

## Supporting information

S1 Table List of species found during surveys. Full list of all 375 species detected during surveys, along with the number of survey points each species was detected at and the number of intervals it was detected during. 215 species were found at a minimum of 5 survey points.
(XLSX)

S2 Table Complete time series of species detections during surveys. The sheet ‘Detection time series’ gives the species recorded for each survey interval. The sheet ‘Locations’ gives the coordinates and altitude for each survey point.
(XLSX)

S3 Table Table of morphological measurements used in analyses. Measures of wing chord, tarsal thickness and beak size come from Price et al 2014; measures of head, body, and overall length come from Rasmussen and Anderton 2005.
(XLSX)

S4 Table Table of congeneric sympatric sister (CSS) species pairs. All CSS pairs where both species were detected at a minimum of 5 survey points, and where both species were found to occur sympatrically in at least one study survey subregion. See the Methods section of the manuscript for a more complete description.
(XLSX)

S1 Tree 1000 phylogenetic tree subset downloaded from the global bird phylogeny dataset associated with Jetz et al 2012, in .tre format. (TRE)

S2 Tree Consensus phylogenetic tree for species in this study. Consensus tree based on the 1000 tree sample given in S1 Tree.
(TRE)
